# Using CT imaging to identify sarcopenia as a risk factor for severe falls in older adults

**DOI:** 10.1186/s12877-025-05707-0

**Published:** 2025-02-01

**Authors:** Nadja Fries, Angeliki Kotti, Mischa Woisetschläger, Anna Spångeus

**Affiliations:** 1 Division of Diagnostics and Specialist Medicine, Department of Health, Medicine and Caring Sciences, Linköping University, Linköping, Sweden; 2 Department of Radiology, Linköping University Hospital, Linköping, Sweden; 3 Department of Biomedical and Clinical Sciences, Linköping University, Linköping, Sweden; 4 Center for Medical Image Science and Vizualization (CMIV), Linköping University, Linköping, Sweden; 5 Department of Acute Internal Medicine and Geriatrics, Linköping University Hospital, Linköping, Sweden

**Keywords:** Sarcopenia, Osteoporosis, Osteosarcopenia, Fall, Body composition, Frailty, Geriatric, Computed tomography

## Abstract

**Background:**

Sarcopenia is a skeletal muscle disease primarily associated with ageing and progressive muscle decline and increases the risk of falls. The purpose of the present study was to investigate risk factors, including sarcopenia, for severe falls compared to non-severe falls. In addition, we wanted to explore possible associations between sarcopenia, bone mineral density (BMD), adipose tissue as well as clinical scores assessing frailty, nutritional status, and fall risk.

**Methods:**

This retrospective cohort study included 101 older patients that had experienced a fall incident during in-patient care at a geriatric ward between 2018 and 2020. The fall incidents were categorized into severe or non-severe falls. Clinical data, including risk assessment scores were retrospectively obtained from the participants’ medical records. Body composition, including skeletal muscle quantity (SKM), adipose tissues, and BMD were assessed from abdominal CT-scans performed for any reason maximal 6 months before or after the fall. Skeletal muscle index ratio (SMI-ratio) was calculated using SKM cm^2^/height m^2^ and divided with previous described cut off values for sarcopenia. An SMI ratio < 100% indicated sarcopenia.

**Results:**

The severe fall group showed higher grade of sarcopenia compared to the non-severe fall group (SMI ratio of 71% vs. 83%, *p* = 0.041) as well as lower, though statistically non-significant, BMI and subcutaneous adipose tissue (SAT) (BMI 22 [20–24] vs. 24 [22–27] kg/m^2^, *p* = 0.108, and SAT 95 ± 70 cm² vs. 141 ± 94 cm², *p* = 0.124). Overweight was more common in non-severe than severe fall group (43% vs. 14%, *p* = 0.048). SMI ratio correlated negatively with frailty and positive with BMI and the following body composition measurements: intramuscular-, subcutaneous, and visceral adipose tissue (IMAT, SAT and VAT). No correlation with other clinical risk assessment scores nor spine T-score was found. In the multivariate analysis, higher level of frailty, male sex as well as lower BMI, VAT and SAT remained as risk factors for low SMI ratio.

**Conclusions:**

These results underscore the importance of addressing sarcopenia and related risk factors, including malnutrition, in the management and prevention of severe falls in the elderly population. Body composition analyzed in CT-scans could add value in this risk assessment. This analysis could be conducted opportunistically during CT scans performed for other purposes.

## Background

Sarcopenia is a progressive skeletal muscle disease that impairs muscle quantity and quality leading to low muscle strength. While primarily associated with ageing and muscle changes that happen over a long period of time, sarcopenia can also occur secondary to e.g. inflammatory processes such as malignancy or organ failure [1].

Today low muscle strength measured with functional tests, is considered to be the main finding in sarcopenic patients [1]. The diagnosis could be further assessed radiologically by the presence of low muscle quantity. A recommended radiological method for assessing lean mass is dual-energy X-ray absorptiometry (DXA) [1]. DXA is advantageous due to its very low radiation dose, which permits multiple reassessments over time without health concerns. However, it necessitates a dedicated machine and a separate visit. Alternatively, sarcopenia and other body composition measures can be identified using CT scans [1], which can be analyzed opportunistically during scans performed for any reason. Given the frequency of performed CT scans nowadays, particularly among the aging population, opportunistic body composition analysis offers a readily available and valuable method for screening sarcopenia in high-risk groups. Compared to DXA, CT provides three-dimensional images and higher resolution, potentially enhancing the accuracy of the analysis.

Previous studies have shown that tissue measurements at the level of lumbar L3 strongly relates to whole body composition and thus one established method is to use the L3 level in computer tomography (CT)-scans as a landmark to calculate muscle cross-sectional area and to predict whole body composition [1, 2]. It thereby enables rapid quantification of different types of tissues such as SKM (skeletal muscle), intramuscular adipose tissue (IMAT), visceral adipose tissue (VAT) and subcutaneous adipose tissue (SAT). This is an easily applicable method as abdominal CT-scans done for other reasons are often available in the patients’ medical records [1–3]. Therefore, if deemed valuable for assessing the risk of severe falls, body composition analysis can be conveniently conducted on routine CT scans (opportunistically) or even in dedicated low-dose CT scans. If an increased risk is identified, appropriate measures can be implemented.

Sarcopenia and osteoporosis share many risk factors as well as negative clinical outcomes such as an increased risk of falls and fractures, frailty, hospitalization and mortality [1, 4–6]. Frailty is a condition with impaired function of multiple physiological systems which consequently predisposes an individual to decreased capacity in maintaining balance and coordination. It also increases the risk for inability to recover from falls [7]. Frailty can be estimated with the Clinical Frailty Scale (CFS) ranging from 1 (very fit) to 9 (terminally ill) [8]. Poor nutrition status is known to fasten the process of developing sarcopenia, and malnutrition with vitamin D deficiency was also shown to be related to balance dysfunction, increasing the risk of falling [1, 9]. A way to estimate nutrition status is by the mini nutritional assessment short-form (SF-MNA) [10]. Poor nutrition status is also a risk factor for developing pressure ulcers. The risk assessment pressure sore (RAPS) scale rates risk factors with an assumption that a lower score generates a greater risk of developing pressure sores [11] .

To clinically identify patients with high risk of falling, the Downton Fall Risk Index (DRFI) is a valuable screening instrument with a score range of 0–11 where 3 or more is considered as a high risk of falls [12]. And lastly the modified elderly mobility scale (M-EMS) is an instrument designed to evaluate mobility and is specially addressed to the older persons. It generates a maximum of 20 points where higher points indicate a more safe and independent movement [13].

Previous studies have shown that same-level falls (low-energy trauma) account for close to half of all traumas in the older persons population and that these patients also pose a higher risk for severe injury compared to the younger population (e.g. head- and neck injuries and fractures) as well as death related to falls [14]. Moreover, osteoporosis combined with sarcopenia is associated with an even higher risk for fracture, but little is known about whether the outcome of a fall can be predicted with the degree of sarcopenia [15, 16].

Compared to non-injurious falls, severe falls pose a significant risk of causing long-term disabilities, such as after fractures and brain injuries. They often necessitate more intensive medical interventions, including hospitalization, surgery, and rehabilitation, and are therefore associated with substantial healthcare costs. Additionally, severe falls can have psychological impacts, such as fear of falling, which may limit patients’ activities [17]. Therefore, a better understanding of the risk factors for severe falls and the identification of protective factors are essential to safeguard the geriatric population.

The aim of this study was to investigate risk factors for severe falls compared to non-severe falls in older persons. In addition, we wanted to explore possible associations between sarcopenia, body composition (bone mineral density [BMD] and adipose tissue) as well as clinical risk assessment scores assessing frailty, nutritional status, and fall risk.

## Methods

### Study design and study cohort

In the present retrospective cohort study, we included all patients at the geriatric acute ward with a fall-incident during an inpatient stay between 1st January 2018 to 31st December 2020 (*n* = 159). Of these patients, only patients with an abdominal CT scan performed within 6 months before or after the fall incident were included (*n* = 101). The fall-incidents were identified through the department’s event tracking system (Synergi). We excluded patients with falls secondary to other clinical events such as stroke, or arrhythmia. Data collected from the patients’ medical record was used to categorize falls into two groups depending on the outcome of the fall, i.e. severe falls and non-severe falls. Severe falls included falls that resulted in fracture, need of opioid prescription, major head injury, internal bleeding, or death, while non-severe falls resulted in no or minor medical conditions (e.g. bruises or laceration with/or without mild analgesics for pain management).

Background data, clinical measures and clinical risk assessment scores were extracted from the medical records. Clinical risk assessment instruments included in the study were CFS, SF-MNA-, DRFI-, M-EMS- as well as RAPS- score. The CFS is an instrument assessing frailty on a 9-point-scale ranging from 1 (very fit) to 9 (terminally ill) [8]. The SF-MNA consists of six questions that cover key areas related to nutritional status and estimates patient’s nutrition status on a 0–14-point scale, where 0–7 point is considered malnourished, and 8–11 at risk of malnutrition [10]. The DRFI is a tool used to assess the risk of falls in patients. Risk is assessed from five modules with a higher score suggesting a greater risk of fall. A score of 3 or more is considered as a high risk of falls [12]. The M-EMS is an instrument designed to evaluate mobility and is specially addressed to older people. It generates a maximum of 20 points where higher points indicate a more safe and independent movement [13]. The RAPS assesses risk factors for pressure ulcers with lower score indicating a higher risk of developing pressure sores [11]. A score *≤* 29 is considered an increased risk of pressure ulcers.

**Fig. 1:**
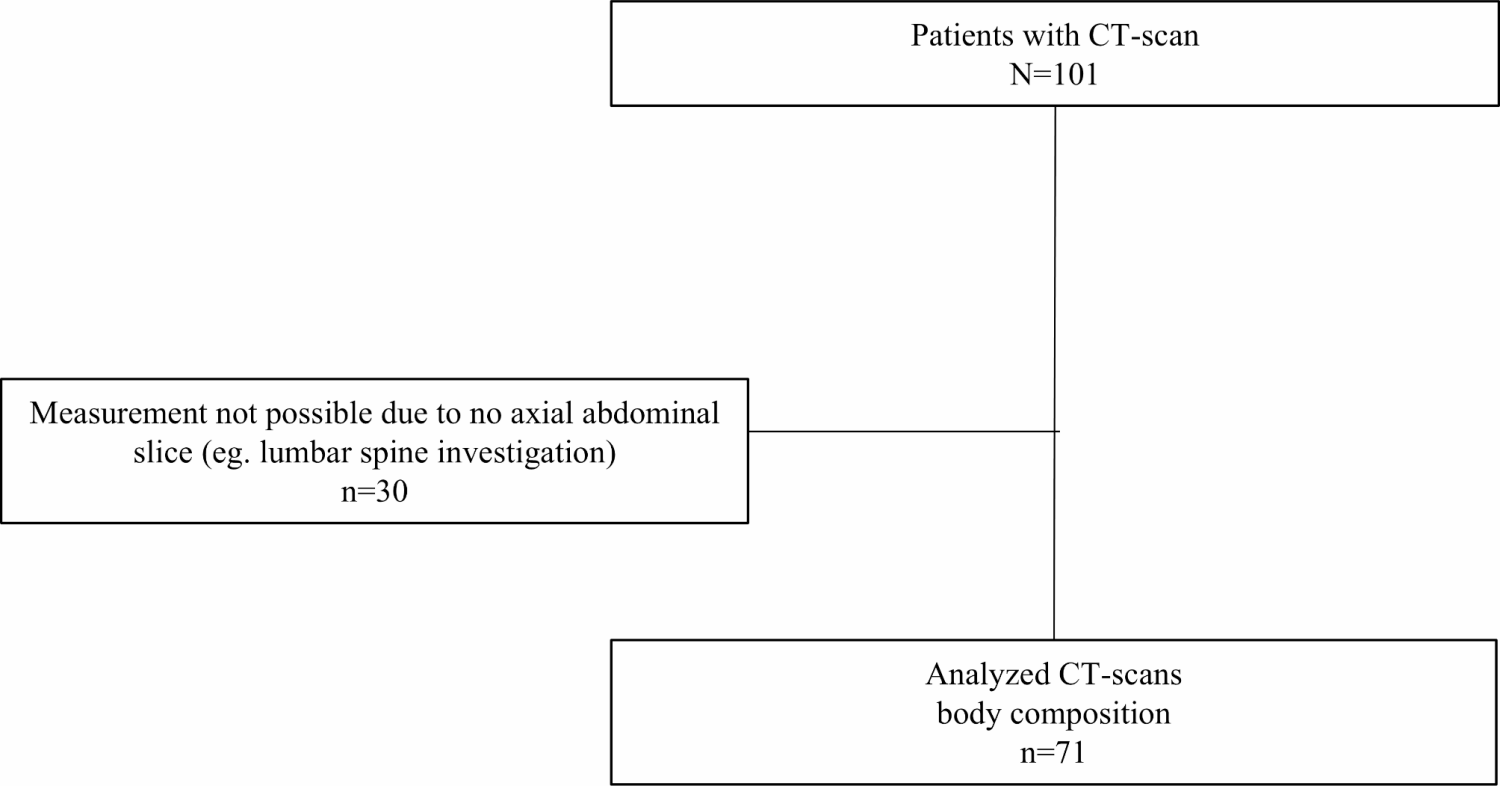
Flow chart of the patient inclusion process for skeletal muscle (SKM) assessment. A total of 101 CT scans were available; however, 30 could not be analyzed due to technical issues, such as incomplete coverage of the abdominal area

For the body composition measurements all abdominal CT-scans performed for any clinical reasons 6 months before or after the reported fall were retrospectively identified using Picture Archiving and Communication System (PACS) (Sectra Imtec AB, Linköping, Sweden, version 25.1) which resulted in 101 unique patients with at least one CT abdominal scan. In the SKM assessment, lumbar spine CT-scans (i.e. scans made with spine protocol) were excluded due to incomplete field of view (*n* = 30). Our final CT cohort consisted of 71 patients with CT-scans available for analysis as shown in Fig. 1. There were no significant differences between patients analyzed with DAFS and those not analyzed, in terms of age (84 ± 8 vs. 84 ± 7 years, *p* = 0.933), sex (46% female vs. 44% female, *p* = 0.873), and the percentage with severe fall trauma (16% vs. 14%, *p* = 0.821). CT scans utilized in the SKM analysis were conducted on average 11 days prior to the fall event (with a median of 6 days). 10% of the scans were performed 3 months or longer after the fall, and 3% were conducted 4 months or longer post-fall.

For BMD assessment, lumbar spine CT-scans and scans with missing calibration data were excluded as they were not possible to analyze in the software (*n* = 44). This resulted in 57 unique patients with CT-scans available for BMD analysis. There were no significant differences between patients analyzed for BMD and those not analyzed, in terms of age (84 ± 8 vs. 85 ± 7 years, *p* = 0.428), sex (47% female vs. 43% female, *p* = 0.622), and the percentage with severe fall trauma (16% vs. 14%, *p* = 0.815).

### Body composition measurements

#### Skeletal muscle

**Fig. 2:**
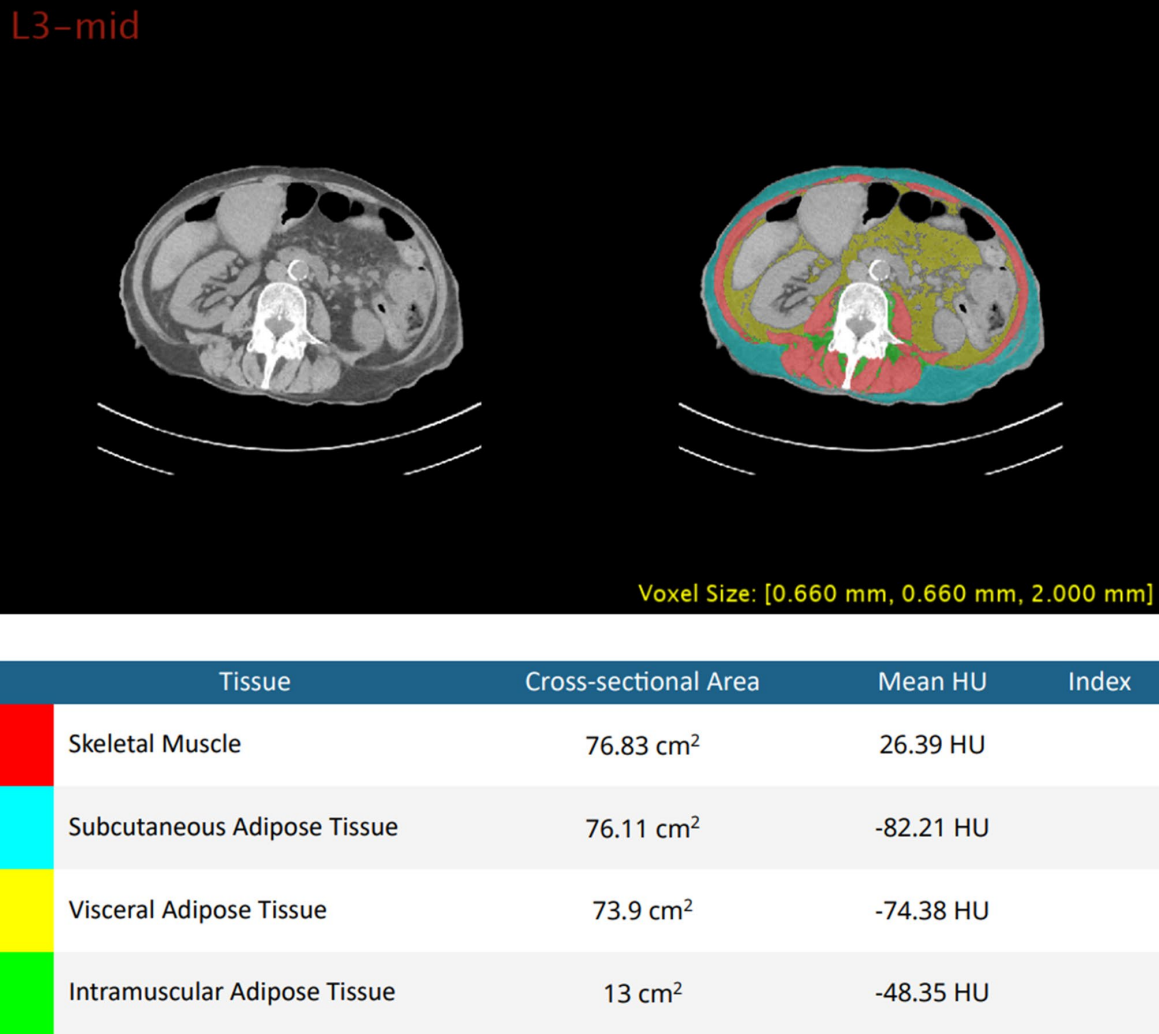
Single-slice body composition measurement with Data Analysis Facilitation Suite (DAFS) (Voronoi Health Analytics, Inc., Vancouver, Canada). The various tissues were automatically identified and analyzed, including skeletal muscles (red) and adipose tissues, which were further sub-categorized into subcutaneous (blue), visceral (yellow), and intramuscular (green)

In each patient´s CT-scan, body composition measurements were obtained by using Data Analysis Facilitation Suite (DAFS) (Voronoi Health Analytics, Inc., Vancouver, Canada). DAFS allows automated single slice as well as multi-slice extraction from CT scans which generates organ segmentation and body composition measurements. For our cohort, single slice SKM, SAT, VAT and IMAT measurements across mid-L3 were calculated and quantified as cross-sectional area in cm^2^ (Fig. 2).

#### Bone mineral density

BMD and T-score were assessed by the Mindways qCT PRO software (version 2, Mindways, Austin, TX, USA). Regions of interest (ROIs) are automatically placed with 7–9 mm thickness depending on how much the shape of the vertebrae allows analysis without distortion of BMD (e.g. compression fracture, posterior venous plexus, cortical bone) in the trabecular part of the lumbar L1-L3 vertebrae and can manually be adjusted if needed. From this, a mean BMD and T-score was calculated. In case T-score varied > 1 SD between vertebrae, we excluded the vertebrae with the highest T-score.

### Defining cut off measurements

#### Skeletal muscle

For the sarcopenia analyses, we calculated the skeletal muscle index (SMI, cm^2^/ m^2^) using the formula: cross-sectional area of SKM divided by the square of body height. Cut off measurements were obtained from a previous study on a healthy Caucasian population with sarcopenia defined as < 41.6 cm^2^/ m^2^ for men and < 32 cm^2^/ m^2^ for women [17]. Each patient’s SMI was divided with these cut off values, resulting in a sex-neutral ratio that represents the participants percent of the cut off value for sarcopenia and is called SMI ratio. In tables and figures SMI ratio is presented in percent.

#### Bone mineral density

T-scores both from the hip and spine (average spine T-score) were used for the analysis.

### Statistical analysis

Descriptives were presented in mean and standard deviation (SD) or median and interquartile range (IQR) for continuous variables according to normal distribution or not, and frequency for categorical variables. For continuous data, group comparisons were made with T-test and Mann-Whitney U according to normality and for categorical data Chi2-test was used.

Possible correlation between sarcopenia with clinical measures and adipose tissue measurements was examined according to normality by Pearson or Spearman.

Univariate linear regression was conducted on variables considered to possibly have an impact on the level of sarcopenia, and statistically significant variables were further analyzed with multiple regression analysis. In the multivariate regression analysis three different models were used; Model (1) sex, outcome of fall, CFS, and BMI; Model (2) sex, outcome of fall, CFS, and SAT; and Model (3) sex, outcome of fall, CFS, and VAT. The models were created due to collinearity between BMI, SAT, and VAT.

All statistical calculations were conducted using SPSS Statistics (version 28.0, IBM Corp., Armonk, NY, USA) and a *p* value < 0.05 was considered significant.

## Results

### Characteristics of the study participants

Cohort characteristics are described in Table 1. Of the 101 participants, 59 were male and 42 female, with a mean age of 84 years (range 62–103 years). In the chosen period a total of 14 (14%) patients had a fall categorized as severe. 84% of the patients were defined as sarcopenic (SMI ratio *≤* 100%). Nearly half of the patients (48%) had sustained previous fractures before the documented fall-incident. The total numbers of fall events during the inpatient stay varied between one and three. Most patients (79%) had only one fall event, 18% had two fall events, and 3% had three fall events. There was no statistical difference in the number of falls between patients categorized as having severe (71% one fall, 29% two falls, and 0% three falls) or non-severe falls (81% one fall, 16% two falls, and 3% three falls).

**Table 1:** Cohort descriptives and group comparisons between non-severe- and severe falls

Variable	All		Non-severe falls	Severe falls	*P*-value severe vs. non-severe falls	
	*n* = 101		*n* = 87	*n* = 14		
**Clinical risk factors**					0.287	
Sex						
male	*n* = 58		83%	17%		
female	*n* = 42		91%	9%		
Age (years)^a^	84 (± 7)		84 (± 7)	85 (± 9)	*0.631*	
Height (cm)^a^	170 (± 10)		170 (± 11)	170 (± 6)	*0.800*	
Weight (kg)^a^	70 (± 17)		71 (± 18)	66 (± 12)	*0.344*	
BMI (kg/m^2^)^b^	24 (21–27)		24 (22–27)	22 (20–24)	*0.108*	
**Medical treatment**						
Blood thinners	63%		68%	79%	*0.183*	
Antihypertensives	69%		68%	71%	*0.787*	
Statins	21%		22%	14%	*0.518*	
Osteoporosis treatment	10%		9%	14%	*0.554*	
FRID-classified medication	93%		92%	100%	*0.262*	
Opioids	39%		40%	29%	*0.695*	
**Medical history before admission**						
Cardiovascular disease	56%		55%	71%	*0.240*	
Diabetes mellitus	27%		26%	29%	*1.000*	
Cognitive Disease	31%		30%	36%	*0.661*	
Parkinson’s disease	13%		12%	21%	*0.383*	
Osteoporosis	20%		21%	14%	*0.577*	
Previous fractures	48%		47%	50%	*0.842*	
**Living situation before admission**					*0.618*	
Own home	31%		29%	43%		
Own home but need of municipal service	56%		59%	43%		
Rehabilitation facility	4%		3%	7%		
Nursing home	9%		9%	7%		
**Physical function before admission**					*0.309*	
Independent	18%		20%	7%		
Walking aids	65%		65%	64%		
Dependent (personal assistance needed)	17%		15%	29%		
**Radiologic assessments**						
T-score spine^a^	-3.9 (± 1.3)		-3.9 (± 1.2)	-3.6 (± 1.4)	*0.419*	
T-score femoral neck^b^	-2.7 (-3.2- -1.9)		-2.7 (-3.3 – -2.2)	-1.9 (-3.2– -1.3)	*0.378*	
SMI ratio in %^a^	81 (± 17)		83 (± 17)	71 (± 14)	***0.041***	
IMAT (cm^2^)^b^	18 (13–23)		18 (7–29)	16 (7–25)	*0.269*	
SAT (cm^2^)^a^	134 (± 91)		141 (± 94)	95 (± 70)	*0.124*	
VAT (cm^2^)^b^	94 (49–220)		96 (54–218)	92 (34–237)	*0.891*	
**Risk assessment score**						
CFS^b^	6 (5–7)		6 (5–7)	6 (6–7)	*0.183*	
SF-MNA^b^	8 (6–10)		8 (5–10)	6 (6–8)	*0.196*	
DFRI^b^	5 (4–6)		5 (4–6)	4 (4–6)	*0.967*	
RAPS^b^	31 (28–32)		31 (28–32)	31 (27–32)	*0.905*	
M-EMS^a^	9 (± 5)		9 (± 5)	10 (± 5)	*0.258*	

Variables presented according to normality, ^a^=mean (SD), ^b^=median (IQR) and categorical in frequency distributions (%); SMI, skeletal muscle index; SKM, skeletal muscle; IMAT, intramuscular adipose tissue; SAT, subcutaneous adipose tissue; VAT, visceral adipose tissue; CFS, clinical frailty score; SF-MNA, Mini Nutritional Assessment - short form; DFRI, Downton Fall Risk Index; RAPS, Risk assessment Pressure Sore scale; M-EMS, Modified Elderly mobility Scale. FRID, fall risk inducing drugs. Blood thinners include both anti-coagulant and platelet inhibitors. Antihypertensives include all classes of antihypertensives. Diabetes mellitus, including type 1- and 2 diabetes mellitus; cardiovascular disease; including coronary heart disease, cerebrovascular disease and peripheral arterial disease; Cognitive Disease, including Alzheimer’s disease, vascular dementia, Lewy body disease, unspecified dementia

Regarding clinical risk assessment scores, 94% of the participants had a high risk of falling according to DFRI (*≥* 3) with a moderate mobility limitation (M-EMS mean score 9 ± 5). 58% were at least moderately frail (CFS *≥* 6). Only 8% of the participants were considered having a normal nutritional status according to the SF-MNA but were at no current risk to develop pressure sores (RAPS median score 31 [28–32]).

### Severe versus non-severe falls

**Fig. 3:**
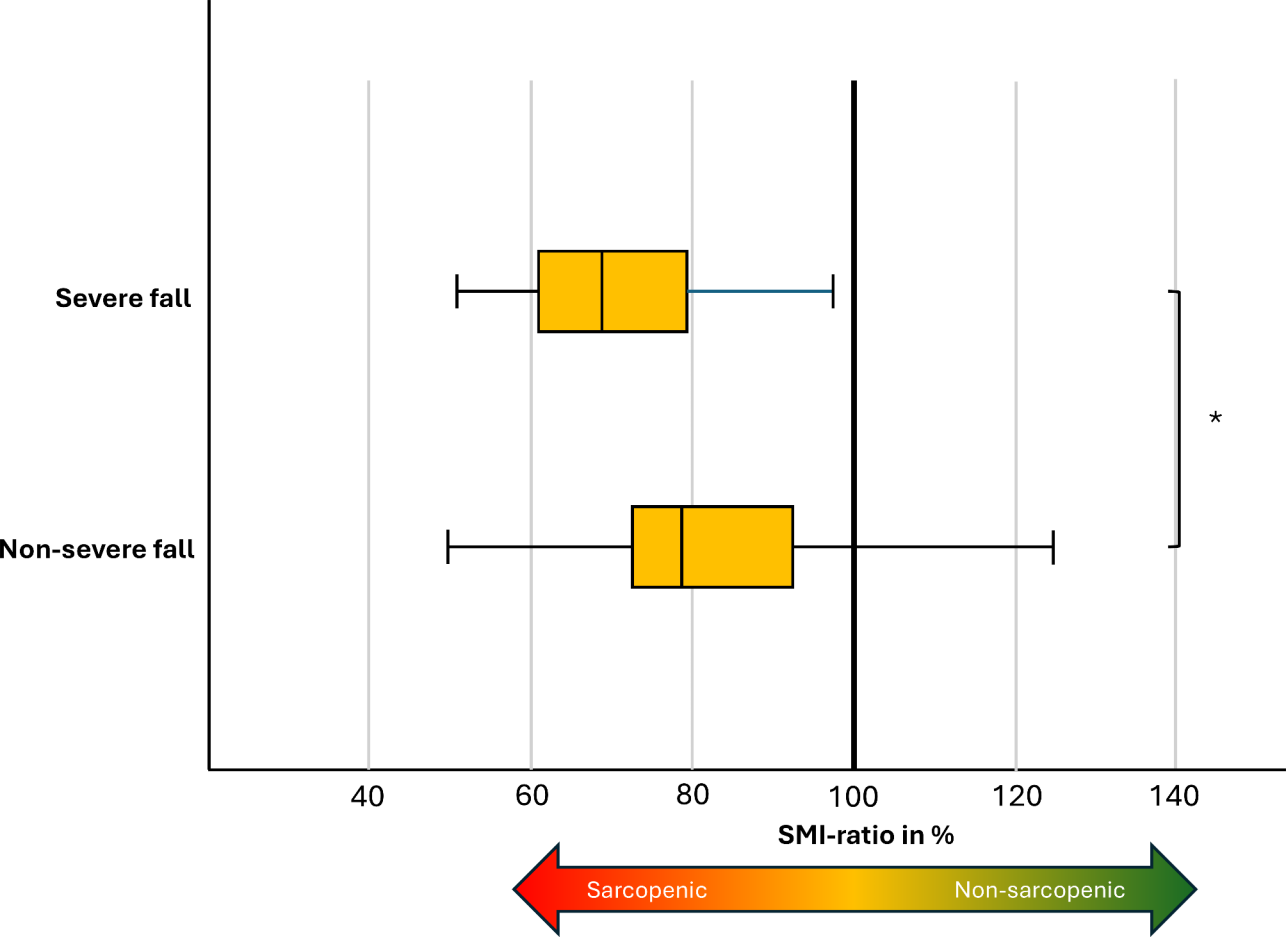
Group comparison between non-severe- and severe falls and skeletal muscle index divided by the cut off measure of sarcopenia, shown in percent (SMI ratio in %). Below 100% is considered sarcopenic

As shown in Table 2, patients with severe falls had significantly lower SMI ratio than patients with non-severe falls (SMI ratio 71% vs. 83%, *p* = 0.041) (Table 2; Fig. 3). Median BMI was lower, though statistically non-significant, in patients who suffered a severe fall compared to the non-severe fall (22 [20–24] vs. 24 [22–27] kg/m^2^, *p* = 0.108). Furthermore, when analyzing BMI categories (underweight < 18.5 kg/m², normal weight 18.5–24.9 kg/m², and overweight ≥ 25 kg/m²), a higher percentage of overweight individuals was observed in the non-severely injured group compared to the severely injured group (43% vs. 14%, *p* = 0.048), reinforcing the protective effect of higher weight. Similarly, subcutaneous adipose tissue (SAT) was nearly 150% higher in non-severe falls compared to severe falls, although this difference was not statistically significant (141 ± 94 cm² vs. 95 ± 70 cm², *p* = 0.124). No statistical difference was seen regarding spine- and femoral neck T-score, body composition measures IMAT and VAT, as well as clinical estimation of frailty (CFS), fall-risk (DFRI), pressure sore (RAPS), mobility (M-EMS) and malnutrition (SF-MNA).

### Associations between Sarcopenia, clinical measures, clinical risk assessment scores and body composition

As shown in Table 2, SMI ratio correlated positively with BMI (rho = 0.438) and adipose tissues (IMAT, SAT and VAT, rho = 0.280, 0.478, and 0.269, respectively), and negative with frailty (CFS, rho = -0.288). There was no significant correlation between SMI ratio with the other clinical measuring scales.

**Table 2:** Correlation between Sarcopenia (SMI ratio) and clinical measures, as well as clinical risk assessment scores

Variable	SMI ratio	*P*-value
Age^a^	-0.150 [-0.384-0.101]	*0.240*
BMI (kg/m^2^)^b^	0.438 [0.206–0.623]	***< 0.001***
Previous fracture^b^	0.152 [-0.107-0.392]	*0.234*
**Body composition**		
T-score spine^a^	0.053 [-0.223-0.321]	*0.710*
T-score femoral neck^b^	-0.032 [-0.345-0.287]	*0.841*
IMAT (cm^2^)^b^	0.280 [0.027–0.499]	***0.026***
SAT (cm^2^)^a^	0.478 [0.261–0.649]	***< 0.001***
VAT (cm^2^)^b^	0.269 [0.015–0.490]	***0.033***
**Clinical risk assessment scores**		
CFS^b^	-0.288 [-0.509 - -0.032]	***0.024***
SF-MNA^b^	0.227 [-0.057-0.477]	*0.106*
DFRI^b^	0.028 [-0.252-0.303]	*0.844*
RAPS^b^	0.104 [-0.188-0.379]	*0.473*
MEMS^a^	0.059 [-0.205-0.314]	*0.665*

^a^= Pearson’s correlation analysis; ^b^= Spearman’s correlation analysis; previous fractures with no coded as 0 and yes coded as 1; SMI, skeletal muscle index; IMAT, intramuscular adipose tissue; SAT, subcutaneous adipose tissue; VAT, visceral adipose tissue; CFS, Clinical Frailty Score; SF-MNA, Mini Nutritional Assessment – short form; DFRI, Downton Fall Risk Index; RAPS, Risk assessment Pressure Sore scale; M-EMS, Modified Elderly Mobility Scale

Univariate- and multivariate regression analyses are presented with SMI ratio as the dependent variable in Table 3. Collinearity was found between weight and height and was therefore presented as BMI (kg/m^2^). Femoral neck T-score was excluded as collinearity was found between spine- and femoral neck T-score. The multivariate regression included all clinical measurements that were significant in the univariate regression but as collinearity was found between BMI, SAT and VAT we analyzed data in three different regression models, as described in methods. Thus, BMI was included in model 1, SAT in model 2, and VAT in model 3.

In the univariate analyses, male sex, severe fall, lower BMI, lower SAT, lower VAT and a higher degree of frailty (CFS) were associated with worse sarcopenia (i.e. lower SMI ratio). In the multivariate analysis, male sex, lower BMI, lower VAT, lower SAT and a higher degree of frailty remained significant risk factors for low SMI ratio.

**Table 3:** Predictive factors of muscle mass (SMI ratio in %), analyzed with univariate- and multivariate linear regression

	Univariate	Multivariate		
		Model 1 *R*^2^ = 0.470	Model 2 *R*^2^ = 0.382	Model 3 *R*^2^ = 0.481
Sex	-10.22 [-18.60- -1.84]*	-12.26 [-19.14- -5.38]*	-8.44 [-15.73- -1.16]*	-16.33 [-23.58- -9.08]*
Age	-0.33 [-0.90-0.23]			
BMI	1.48 [0.83–2.14]*	1.56 [0.97–2.14]*		
Outcome of the fall	-11.61 [-22.71- -0.51]*	-4.72 [-13.65-4.22]	-4.88 [-14.59-4.83]	-5.93 [-14.71-2.85]
Previous fracture	4.27 [-4.40-12.93]			
T-score spine	0.68 [-2.95-4.30]			
IMAT (cm^2^)	0.37 [-0.07-0.81]			
SAT (cm^2^)	0.09 [0.05–0.13]*		0.08 [0.04–0.12]*	
VAT (cm^2^)	0.05 [0.02–0.09]*			0.08 [0.05–0.11]*
CFS	-3.82 [-7.17- -0.48]*	-3.20 [-5.83–0.57]*	-3.42 [-6.25- -0.58]*	-3.20 [-5.80- -0.60]*
SF-MNA	1.56 [-0.20-3.32]			
DFRI	0.39 [-3.15-3.93]			
RAPS	0.55 [-0.94-2.04]			
MEMS	0.20 [-0.72-1.11]			

*p < 0.05; SMI, skeletal muscle index; outcome off the fall with non-severe fall coded as 0 and severe fall coded as 1; sex with female coded as 0 and male coded as 1; previous fractures with no coded as 0 and yes coded as 1; IMAT, intramuscular adipose tissue; SAT, subcutaneous adipose tissue; VAT, visceral adipose tissue; CFS, Clinical Frailty Score; SF-MNA, Mini Nutritional Assessment – Short form; DFRI, Downton Fall Risk Index; RAPS, Risk assessment Pressure Sore Scale; M-EMS, Modified Elderly Mobility Scale

All three multivariate models included sex + outcome of fall + CFS. In addition to this model 1 included BMI, model 2 included SAT, and model 3 included VAT

## Discussion

In the present study, we showed that patients who experienced severe falls exhibit more pronounced sarcopenia compared to non-severe fallers. Additionally, there was a trend indicating that severe fallers had lower BMI and reduced subcutaneous adipose tissue. A greater severity of sarcopenia was associated with male sex, lower adipose tissue levels, lower BMI, and frailty.

A previous Italian study with a similar cohort as ours with a mean age of 86 years suggested that sarcopenia increases the risk of falling up to three times in two years follow up, yet little is known whether the outcome of the fall was associated with the degree of sarcopenia [18].

In our study, participants were on average moderately frail with a median of 6 on the CFS and had all sustained a fall during their stay at the geriatric ward. Most patients (84%) had a SMI ratio below 100%, indicating sarcopenia. SMI ratio differed significantly between the groups where those who sustained a severe fall had more severe sarcopenia compared to the non-severe fall group (SMI ratio of 71% vs. 83%, *p* = 0.041). In contrast to our finding, a study on Swedish women aged 75–80 years reported that sarcopenia based on definitions including low muscle mass, i.e. lean mass assessed by DXA (European Working Group on Sarcopenia in Older People [EWGSOP2] [1], and Asian Working Group for Sarcopenia [AWGS] [19]) was not associated to severe falls, whereas sarcopenia defined using only functional tests (Sarcopenia Definitions and Outcomes Consortium, SDOC) was [16]. The discrepancies between our study and the other study may be attributed to several factors. Firstly, the patient cohorts differ in terms of age (our cohort had a higher mean age, 84 years compared to 78 years), sex (our study included both sexes, whereas the other study included only women), and functional status (only 18% of our participants could walk without assistance, compared to all participants in the other study). Furthermore, the study designs differ: our study uses case reports to define and categorize the severity of each fall, comparing severe to non-severe falls, and utilizes CT scan data from a near time frame, i.e., within six months of the fall, for sarcopenia assessment. In contrast, the other study defines fall injuries based on ICD-coded fall events over a seven-year follow-up period post-sarcopenia assessment. The methodologies also differ in terms of technique; our study employed 3-D CT scans, while the other used 2-D DXA.

A low BMI is associated with a higher risk of sustaining a fall (non-injurious or not) [20]. Little is known about the risk of severe falls. In our study, we saw a trend towards protective effect regarding fall severity with higher amount of adipose tissue, especially SAT, with a mean of 95 ± 70 cm^2^ in the severe fall group compared to 141 ± 94 cm^2^ in the non-severe fall group. Although both groups are considered normal weight on average according to BMI, BMI was higher in the non-severe fall group (BMI 22 kg/m^2^ vs. 24 kg/m^2^ in the non-severe fall group) as was the percentage of overweight patients (43% vs. 14%). Collectively, these findings suggest a protective role of adipose tissue, underscoring the importance of nutritional interventions in this population to prevent severe injurious falls. Consistent with our results, Ek et al. identified underweight as a risk factor for injurious falls [21].

Measurement of body composition using CT-scans could provide an easy and valuable method for assessing sarcopenia and nutritional status in the geriatric population. An automated software solution, like the one used in the present study [22], could offer a rapid and accurate tool for assessing body composition even from opportunistic CT scans, i.e. CT scans performed for any reason. In this case, no additional visits or scans are required, thereby keeping the assessment costs low. Considering that CT investigations are frequently performed in aging populations, they could offer an excellent opportunity to evaluate body composition, including muscle, adipose tissue, and bone in a large-scale screening.

In our study, two different software solutions were used that both are reliable tools providing trustworthy body composition measurements. Until now radiological methods are mainly used for research [2, 23].

In our study, males exhibited a higher risk of developing sarcopenia compared to females. This finding remained significant even after adjusting for nutritional status (BMI, SAT, and VAT). Several previous studies have reported similar results, indicating a higher prevalence of sarcopenia in males than in females [24–26]. The underlying reasons are unclear and likely multifactorial, potentially involving hormonal differences between the sexes [25]. Regarding sex differences in severe falls, a previous study by Aryee et al. found a higher risk of severe falls in males than in females [27]. Similarly, in our study, nearly twice as many males as females experienced a severe fall (17% vs. 9%).

Interventions to improve skeletal muscle function to treat sarcopenia may also treat frailty as they both seem to lead to reduced muscle strength. Studies also suggest that sarcopenia is a risk factor for developing frailty [15]. Interestingly, no difference was seen regarding frailty (CFS) between severe and non-sever falls, but CFS correlated with SMI ratio (*r*=-0.288, *p* < 0.05). The univariate regression analysis showed similar results where every step up on the clinical frailty scale led to a significant decrease of SMI ratio of 3.8%. CFS remained significant even when adjusted for other variables in the three multivariate models, suggesting frailty as an independent risk factor for sarcopenia or vice versa.

Poor nutrition status is known to fasten the process of developing sarcopenia [1, 9]. In our study, however, no association was found regarding sarcopenia and SF-MNA or RAPS. Similarly, those risk assessment scores did not differ between severe and non-severe falls.

Even though DRFI is a valuable screening instrument for fall, it does not consider the severity of a fall. In accordance, our study showed no association between DFRI score and risk of severe falls.

The European Working Group on Sarcopenia in Older People (EWGSOP) recommends the use of SARC-F questionnaire (acronym for strength, assistance walking, rise from a chair, climb stairs, and falls) as a first step to screen for sarcopenia in health care as it has a high specificity to predict low muscle strength [1]. Unfortunately, this questionnaire was not used in our clinic. Similarly, no functional tests, such as grip strength or low gait speed, were used regularly in clinical routine.

A guideline published 2018 from the task force of the International Conference on Sarcopenia and Frailty Research (ICSFR) advocate that the treatment of sarcopenia should consist of physical activity as well as a protein-rich diet or protein supplementation [28].

Even so, sarcopenia is not always equal to low fat mass, it can also present itself as sarcopenic obesity and this is more common in the geriatric population [1]. In this phenotype fat infiltration usually can be found in muscles, leading to a poorer muscle function. This is contrary to the case of malnutrition where low adipose tissue is often present [1]. In our cohort there was a small difference in IMAT between the severe fall- and the non-severe fall group, however non-significant. Increased adipose tissue correlated well with increased muscle mass where SAT was prominent which is in accordance with the assumption that our cohort is at risk of malnutrition with a median of 8 on the SF-MNA.

### Limitations

There are some limitations in our study. The number of observations in this study is low, with risk of underpower and falsely non-significant results. Many patients were lost due to CT scans that were not compatible with the DAFS software.

As a retrospective cohort study design, it’s at risk for several research biases. Even though the clinical assessment scales are well validated and reliable tools there may be risks of observer bias. Furthermore, there is a risk that some fall events were poorly or vaguely described in the case records, potentially leading to misclassification in the study. To address this, we conducted a comprehensive evaluation of each patient case, utilizing all available case record data including input from all professionals in the ward.

Although most CT scans were conducted shortly before the fall event (with a median of 6 days before the fall), some patients had their CT scans several weeks post-incident. In these cases, low muscle mass could have resulted from immobilization following the fall, rather than pre-existing low muscle mass that increased the risk of a severe fall. However, the number of CT scans performed more than three months after the fall was low (7 scans, or 10%), likely having low impact on the overall data. Strengths of the study included the design that all data was collected from the same ward, with routines for reporting clinical assessment scores for all patients, and well-defined reporting system regarding fall incidents and severity.

## Conclusions

The conclusion of this study is that older patients who suffer a severe fall have less skeletal muscle quantity measured by CT-scan compared to patients that get mildly injured in the fall. Adipose tissue appears to have a protective effect in the event of a fall and in preventing sarcopenia, with subcutaneous adipose tissue providing the most significant protection. Therefore, diagnosing and treating sarcopenia and malnutrition may be crucial in reducing the severity of fall-related injuries in geriatric patients. An integrated assessment of muscle and adipose tissue in CT scans, e.g. in opportunistic screening or dedicated low dose CT scans, should thus be considered for future geriatric assessment. Once identified, preventive measures should be implemented to improve muscle and nutritional status.

## Data Availability

The datasets generated and analyzed during the current study are not publicly available but are available from the corresponding author on reasonable request.

## Abbreviations

BMD: Bone mineral density
SKM: Skeletal muscle
CT: Computed tomography
SMI: Skeletal muscle index
CFS: Clinical frailty score
BMI: Body mass index
IMAT: Intramuscular adipose tissue
SAT: Subcutaneous adipose tissue
VAT: Visceral adipose tissue
SARC-F: Acronym for strength, assistance walking, rise from a chair, climb stairs, and falls)
DXA: Dual energy x-ray
MRI: Magnetic resonance imaging
SD: Standard deviations
SF-MNA: Mini nutritional assessment - short form
RAPS: Risk assessment pressure sore
DFRI: Downton fall risk index
M-EMS: Modified elderly mobility scale
PACS: Picture archiving and communication system
DAFS: Data analysis facilitation suite
ROI: Regions of interest
IQR: Interquartile range
EWGSOP: The european working group on sarcopenia in older people
ICSFS: International conference on sarcopenia and frailty research
